# Technical and functional outcome after sacral neuromodulation using the “H” technique

**DOI:** 10.1007/s00508-022-02115-x

**Published:** 2022-12-06

**Authors:** Mohammad Mahdi Kasiri, Martina Mittlboeck, Christopher Dawoud, Stefan Riss

**Affiliations:** 1 Division of General Surgery, Department of Surgery, Medical University of Vienna/AKH, Waehringer Guertel 18–20, 1090 Vienna, Austria; 2 Section for Clinical Biometrics, Center for Medical Statistics, Informatics, and Intelligent Systems, Medical University of Vienna, Vienna, Austria

**Keywords:** Neurostimulation, InterStim, Fecal incontinence, Constipation, H technique

## Abstract

**Background:**

Sacral neuromodulation (SNM) is a widely accepted treatment for pelvic floor disorders, including constipation and fecal incontinence (FI). In 2017, a standardized electrode placement method, the H technique, was introduced to minimize failure rates and improve clinical outcomes. We aimed to investigate the technical feasibility and functional outcome of the procedure.

**Methods:**

In this prospective study, we evaluated the first 50 patients who underwent SNM according to the H technique between 2017 and 2020 at a tertiary care hospital. Patient demographic and clinical data were collected, and the impact of various factors on patients’ postoperative quality of life (QoL) was assessed after a follow-up of 40 months. Functional outcome was monitored prospectively using a standardized questionnaire.

**Results:**

Of 50 patients, 36 (72%) reported greater than 50% symptom relief and received a permanent implant (95% CI: 58.3–82.5). We observed 75% success in relieving FI (95% CI: 58.9–86.3) and 64% in constipation (95% CI: 38.8–83.7). Complication occurred in five (10%) patients. Preoperative vs. postoperative physical and psychological QoL, Vaizey score, and obstructed defecation syndrome (ODS) scores revealed significant improvements (all *p* < 0.01). Male gender was significantly associated with postoperative complications (*p* = 0.035).

**Conclusion:**

We provide evidence for the technical feasibility and efficacy of the SNM implantation using the H technique. The medium-term results are promising for patients with FI and constipation. Male patients and those with a BMI > 25 are more prone to perioperative complications.

## Introduction

Sacral neuromodulation (SNM) is a widely accepted treatment for patients with functional pelvic disorders that do not adequately respond to conservative treatment. The complication rate is low, and only a moderate number of patients require a device explantation due to infection, pain, or dysfunction [1].

The SNM involves electrical stimulation of peripheral nerve roots, primarily the sacral spinal nerve S3. The four electrodes use continuous low-intensity electrical pulses to modulate neuronal responses [2]. The implantation process is commonly performed in two successive steps. The first step is a diagnostic trial requiring 2–3 weeks to test the patient’s response to the treatment. If the patient responds positively, the generator is implanted permanently.

Patient selection is certainly important, although clear parameters to select the ideal candidate for neuromodulation are not well defined. In addition, it is commonly speculated that technical factors may also contribute to improved functional outcome. Recently, it has been noted that the close proximity of the implanted electrodes to the targeted neural roots leads to better clinical outcomes [2].

Matzel et al. introduced the “H” technique to optimize electrode placement and minimize the treatment failure rate. The technique requires fluoroscopic identification of the medial edges of the sacral foramina and the inferior end of the sacroiliac joint. An “H” is drawn on the skin site, and the intersection points of the “H” are intended as entry sites [2]. The “H” technique promises a better neuromodulatory effect by ensuring a close proximity of the electrode leads to the targeted sacral root S3 as we demonstrated in a previous study [3].

The present study investigated the technical feasibility and functional outcome of this novel implantation technique by measuring success, failure, and complication rates in patients treated for severe fecal incontinence (FI) and constipation.

## Material and methods

In this prospective single-center study in a tertiary care hospital, we analyzed the first 50 patients between July 2017 and November 2020 who underwent SNM therapy using the “H technique.” All included patients were diagnosed with constipation or FI and FI was defined as the involuntary loss of solid or liquid stool or gas and further assessed by the Vaizey incontinence score [4]. Causes of incontinence included posttraumatic, idiopathic and multifactorial origins. The posttraumatic group comprised patients with low anterior resection syndrome (*n* = 12, 63%), sphincter injuries (*n* = 5, 27%), and other anal surgeries (*n* = 2, 10%). Constipation was defined by the Rome III criteria [2, 5]. Patients were chosen for SNM treatment if conservative treatment strategies had failed. These included lifestyle and diet changes, pelvic floor exercise, unresponsive to laxatives and prokinetics. The application of SNM treatment in each patient population was according to the international guidelines published by Goldman et al. [6]. All operations were performed or supervised by a single colorectal surgeon. Demographic data and overall pelvic function were recorded for all patients using a standardized questionnaire at baseline and during the last follow-up.

Bowel function was evaluated in more detail by the obstructed defecation score, which ranged from 0 (no symptoms) to 31 (very severe symptoms) [7]. The severity of FI was assessed using the Vaizey incontinence score: 0 points indicates no incontinence and 24 points the worst incontinence [4]. Preoperative and postoperative evaluation of a patient’s quality of life was assessed using the SF-12 survey, which includes a mental component score (MCS-12) and a physical component score (PCS-12) [8]. Evaluation of QoL regarding urinary incontinence was assessed by the international consultation on incontinence urinary incontinence short form (ICIQ-SF), using a score scale of 0–21 [9]. Over 50% improvement of all scores between the baseline and the last follow-up visit was considered a successful outcome.

Patients subjected to SNM treatment underwent a two-step procedure. First, diagnostic stimulation was conducted over 14–21 days. Over 50% of self-reported improvement from the baseline condition was considered a positive criterion for permanent implantation of the neurostimulator. Complications and re-operations were assessed after up to 40 months of follow-up. All patients received an InterStim SNM device (Medtronic, Minneapolis, MN, USA).

### The “H” electrode placement technique

**Fig. 1:**
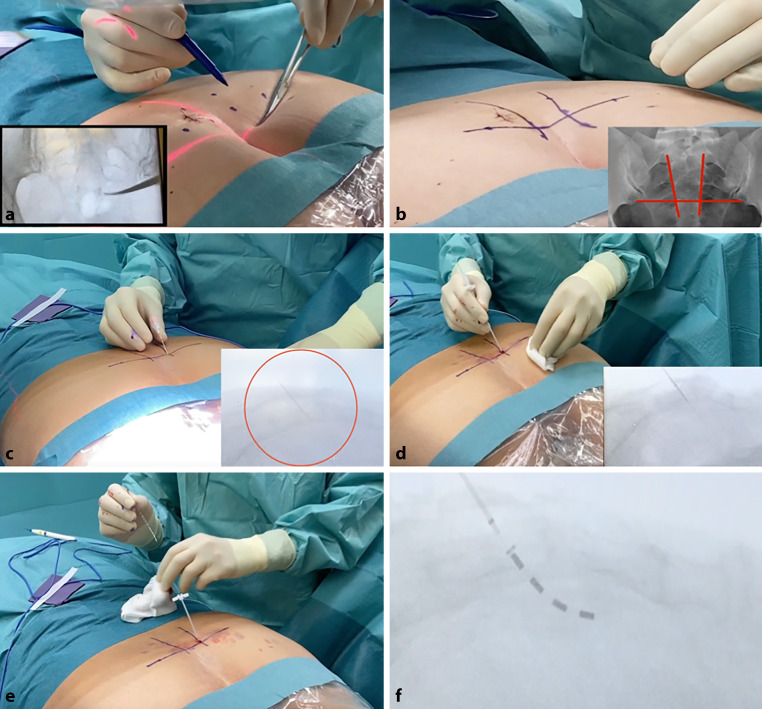
The “H” technique for percutaneous electrode placement is presented. The patient is placed in prone position. **a** An X‑ray of the sacrum helps to locate the landmarks that guide needle placement. The landmarks are the sacral foramina **a**,**b** and the sacroiliac joint. **b** The vertical lines connecting the medial edges of the foramina and the horizontal line connecting the lower edges of the sacroiliac joint produce an “H”. The lines are marked on the skin and the intersecting points represent the ideal entry point for placement of the needle in S3. **c** The needle is then advanced, with lateral X‑rays used for guidance. As presented by X‑ray marked by *red circle*. **d** A guidewire is introduced over the needle followed by a dilatator after the needle is removed. **e** The electrode is placed through the dilatator

The “H” technique for percutaneous electrode placement has been standardized to minimize failed treatment attempts due to technical shortcomings [2]. For the procedure, the patient is placed in the prone position, with the pelvis supported to minimize lumbar lordosis. An X‑ray of the sacrum then helps locate the landmarks, the sacral foramina and the sacroiliac joint that guide needle placement. The vertical lines connecting the medial edges of the foramina and the horizontal line connecting the lower edges of the sacroiliac joint produce an “H.” These lines are marked on the skin, and the intersecting points represent the ideal entry point for needle placement in S3. The needle is then advanced, with lateral X‑rays used for guidance and minor positioning corrections. The needle placement is tested with stimulations to maximize the response. This stimulation can also elicit characteristic movements, depending on the sacral foramen. Under fluoroscopic control, the needle is then replaced by a guidewire and a dilatator, followed by the electrode (Fig. 1).

### Statistical analysis

Absolute and relative frequencies describe categorical variables. The corresponding 95% confidence intervals (95% CI) were computed according to the method of Wilson [10]. The association between two categorical variables was tested by a χ^2^-testor Fisher’s exact test for small group sizes. Continuous variables are described by median, minimum, and maximum based on their skew distributions. Furthermore, the impact of various factors, including sex, BMI, diagnosis, age, and comorbidities on the success rate of SNM treatment were analyzed by logistics regression. The Wilcoxon signed-rank test assessed comparisons between preoperative and postoperative scores. All statistical calculations were performed without data imputation. For all tests, a two-sided *p*-value of ≤ 0.05 was considered significant. Data obtained were evaluated statistically using IBM SPSS Statistics (version 25, SPSS Inc., Chicago, IL, USA).

### Ethics approval

This study was approved by the ethics committee of the Medical University of Vienna (EK Nr. 1267/2020) and conducted according to the principles of the Helsinki Declaration and good clinical practice.

## Results

Out of 50 patients 36 (72%) presented over 50% symptom improvement during the test stimulation period and received a permanent SNM implantation (95% CI: 58.3 and 82.5). The success rate was 75% among patients with FI (27 of 36 patients; 95% CI: 58.9 and 86.3) with the measured stimulation amplitude of 1.7 V (0.8–6.7 V) and 64% in patients with constipation (9 of 14 patients; 95% CI: 38.8 and 83.7) with the measured stimulation amplitude of 2 V (0.4–2.9 V). The treatment benefits were not significantly different between the two patient populations (*p* = 0.4955) and no differences have been observed concerning battery life since no battery explantation was necessary in either group. In 12 (24%) patients not adequately responding to SNM treatment, the leads were removed immediately after the test period and patients were either treated conservatively or underwent additional surgical procedures. Patient characteristics are presented in Table 1.

**Table 1:** Patient demographic data and medical characteristics

**Age** (years)	Median 68 (range 21–92)
**Total number of patients**	50 (100%)
**Ethnicit**y	Caucasian (100%)
**Sex**	
*Female* *Male*	41 (82%) 9 (18%)
**BMI**	Median 25 (range 13–42)
**Patients with comorbidities**	36 (72%)
**Diagnosis**	
*Fecal incontinence* Female Male *Constipation* Female Male	36 (72%) 28 (78%) 8 (22%) 14 (28%) 13 (93%) 1 (7%)
**Cause of incontinence**	
*Posttraumatic* *Idiopathic* *Multifactorial*	19 (38%) 26 (52%) 5 (10%)
**Cause of constipation**	
*Slow transit constipation* *Obstructed defecation syndrome*	7 (14%) 7 (14%)
**Patients with comorbidities**	36 (72%)
*Cardiac disorders* *Endocrine disorders* *Oncologic disorders* *Pulmonary disorders* *Psychiatric disorders*	24 (48%) 11 (22%) 18 (36%) 4 (8%) 5 (10%)

We observed 5 (10%) complications after surgery. Two (4%) infections and one (2%) subcutaneous hematoma were treated conservatively successfully. In 2 (4%) patients, pain at the generator site led to the explantation of the SNM device. Data are presented in Table 2.

**Table 2:** Treatment success and complications of SNM treatment

**Follow-up time (months)**		
Overall Fecal incontinence Constipation	Median 14.2 (Range 0.4–64.8) Median 28.5 (Range 5.5–60) Median 14 (Range 0.4–64.8)	
**Overall success** *Fecal incontinence* *Constipation*	36 (72%) 27 (75%) out of 36 9 (64%) out of 14	
Male Female	6 (66.7%) out of 9 30 (73.2%) out of 41	
**Complication** *Fecal incontinence* *Constipation*	5 (10%) out of 50 4 (11%) out of 36 1 (7%) out of 14	
Male Female	3 (33.3%) out of 9 2 (4.9%) out of 41	
*Sensory*	2 (4%)	1 × Clavien D. II
		1 × Clavien D. IIIb
*Infection*	2 (4%)	1 × Clavien D. II
		1 × Clavien D. IIIb
*Local reaction/Hematoma*	1 (2%)	1 × Clavien D. II
*Lead dislocation*	0	
*Urinary retention*	0	
Further surgical treatment of nonresponder	3 × Sphin-keeper 1 × Colporrhaphy 2 × Rectopexy	

### Functional outcome and quality of life

Patients with FI and constipation showed a significant improvement in both scores. (Vaizey and ODS scores, *p* < 0.0001). The median Vaizey score improved by 6 points. The median ODS score of the 24 patients with ODS > 0 presurgery improved by 3.5 points. Data are provided in more detail in Table 3.

**Table 3:** Preoperative vs. postoperative evaluation of patient quality of life

Life quality eval. (SF 12)	*n*	Pre-op median (min–max)	Post-op median (min–max)	Diff (Pre-post) median (min–max)	*p*-value
*Physical score* *Mental score*	46 50	39 (22–56) 37 (30–59)	50 (38–59) 55 (31–66)	−13 (−32–15) −11.5 (−31–24)	< 0.0001 < 0.0001
**Vaizey incontinence score**	50	16 (0–22)	2 (0–22)	6 (−6–19)	< 0.0001
**ODS score**^ab^	24	11 (1–24)	3 (0–23)	3.5 (0–20)	< 0.0001
**Urinary incontinence score**^b^	18	10 (1–19)	4 (0–15)	6 (0–18)	0.0005

The SF-12 survey is a general health questionnaire containing a mental component score (MCS-12) and a physical component score (PCS-12), Vaizey incontinence minimum score = 0 (perfect continence), maximum score = 24 (total incontinence), ODS score: minimum score 0, maximum score 31, ICIQ-SF: minimum score 0, maximum score 21

^a^Obstructed defecation syndrome

^b^Only patients with score > 0 preoperative

The median urinary function of the 18 (36%) patients who complained about associated urinary incontinence prior to SNM implantation significantly improved by 6 points at the follow-up visit (*p* = 0.0005).

Physical and psychological QoL increases after SNM implantation were highly significant (*p* < 0.0001 for both), with median increases of 13 and 11.5, respectively. Out of 50 preoperative physical QoL data 4 (8%) were missing due to a documentation error. Data are presented in Table 3.

Notably, no parameter predicted significant postoperative functional success. Regarding complications following SNM implantation, male gender with 3 complications of 9 male patients (33%) had a significantly higher risk compared to females (2 complications in 41 patients; 4.9%; *p* = 0.035). Furthermore, patients with BMI ≤ 25 had less complications (1 of 30 patients, 3.3%) compared to patients with BMI > 25 (4 of 20 patients; 20%; *p* = 0.0686).

## Discussion

Our study provides compelling evidence of the technical feasibility of the SNM implantation using the “H technique,” a standardized electrode placement technique initially introduced by Matzel et al. in 2017 [2]. This technique was established to standardize the procedure and to place the electrode as close as possible to the sacral nerves. As a consequence it could be speculated that this leads to improved functional results and increased patient acceptance. Notably, to the authors’ knowledge little is known whether this assumption is valid and patients do benefit from this approach. Based on a recent cadaver study conducted by Müller et al. [3], the “H technique” ensures close proximity of the electrodes to the nerve roots. The authors found that the median distance of the electrodes to the sacral nerve was 0 mm for the most proximal, 0.5 mm for the second, 2.25 mm for the third and 1.75 mm for the most distant electrode. These results support its use and the assumption that better clinical outcomes can be expected.

The occurrence of only 5 minor perioperative complications after a midterm follow-up of up to 40 months indicates the procedure’s safety. Complications occurred more frequently among patients with FI 4 (11%) than constipation 1 (7%). These results contrast with those of Hidaka et al., in which complications were experienced by 10 (12.5%) FI patients and 7 (17.5%) constipated patients. The difference might be due to the low number of patients included in our study [11]. We managed the 4% of patients with infection conservatively, which is consistent with infection and treatment data presented by Wexner et al. (4.1%) [12]; however, none of our patients required surgical intervention due to infectious complications.

During the past years technical advances allowed more precise electrode placement and improved clinical outcome. Among many implantation techniques introduced so far, Amoroso et al. [13] demonstrated the computed tomography guidance for needle and electrode positioning with 60% (18/30) success in patients with pelvic disorders. Adelstein et al. [14] demonstrated 89.0% (113/127) success in patients with urinary incontinence after using the optimal staged lead placement technique using a curved stylet, described by Liberman et al. [15] in 2016. In this technique the electrode is placed medially through the superior aspect of the S3 foramen, and curves laterally. In the fluoroscopy the lead is displayed superior and parallel to the S3 fusion joint. Another previous study described the use of the percutaneous technique for SNM electrode implantation, which resulted in 69% (22/32) success [16]. In this technique electrode leads are placed into the desired sacral foramen through a needle-guided metal stylet. Only a tiny skin incision is necessary to allow the anchor fixation; however, using the “H” technique, we observed 72% (36/50) overall success supporting the implementation of the procedure. Noteworthy, several factors may contribute to a successful outcome, thus making the comparison even more difficult.

Accumulated data support the efficacy of SNM treatment in patients with FI, but the success of treatment in constipated patients is still unclear [17, 18]. Recent studies demonstrated promising short-term outcome for patients with constipation and declining therapeutic effects down to 30% after a long-term period [19, 20]. Knowles et al. showed high response rates to SNM treatment in patients with constipation and rectal hyposensitivity [21]. These results might help to further improve the selection process in this group of patients. Others indicated SNM as an ineffective treatment in patients with constipation [22]. Yiannakou et al. showed a strong placebo response in patients with constipation after treatment with SNM [23]; however, according to our observations, patient self-reported QoL increased significantly in both physical and psychological components in both patient populations. In addition, patients self-reported clinical condition in terms of overall satisfaction also improved significantly in both groups. This indicates that both patient populations benefitted similarly from SNM treatment implanted using the “H” technique; however, whether these patients with constipation benefit from SNM in the long term is unclear and cannot be assessed definitely.

Among 41 (82%) female and 9 (18%) male patients included in our study, there were no significant gender-specific differences regarding baseline information or preoperative vs. postoperative QoL; however, we observed a higher success rate in women (83%) and more postoperative complications in men (60%). These observations contrast with those of Meng et al. [24], which indicated minor QoL improvement and a lower success rate in women, and no significant difference in postoperative complications between the two genders. We propose that both genders may benefit from the treatment and demonstrate a positive risk/benefit profile. Furthermore, similar to Schönburg et al. [25], our data show that age does not play a decisive role in SNM treatment regarding postoperative success and QoL. Finally, regarding patient weight, Marcelissen et al. [26] also found that deriving benefit from SNM treatment is independent of patient weight; however, we also provide insignificant evidence that overweight patients (BMI > 25) are more likely to experience perioperative complications.

There are a few limitations of this study that need to be addressed. We included patients with both fecal incontinence and constipation, creating an inhomogeneous group of patients; however, we considered it essential to assess the outcome of this novel technique for both indications. We conducted a single-arm study, therefore comparison with other techniques can be complex and interpretation needs to be done with caution.

## Conclusion

In this prospective study, we provide evidence about the technical feasibility, safety, and efficacy of the SNM implantation using the “H” technique. The medium-term results are promising for patients with FI and constipation; however, whether this surgical technique is superior to conventional techniques needs to be clarified by further studies. One clear advantage of the “H” procedure is the well-structured surgical approach that can be simply imparted and demonstrated [27].
